# Cytokines in Cancer Immunotherapy

**DOI:** 10.3390/cancers3043856

**Published:** 2011-10-13

**Authors:** Sylvia Lee, Kim Margolin

**Affiliations:** 1 Division of Medical Oncology, Department of Medicine, University of Washington, Seattle, WA 98195, USA; E-Mail: smlee@fhcrc.org; 2 Clinical Research Division, Fred Hutchinson Cancer Research Center, Seattle, WA 98109, USA

**Keywords:** cytokines, cancer, immunotherapy, interleukin-2, interferon

## Abstract

Cytokines are molecular messengers that allow the cells of the immune system to communicate with one another to generate a coordinated, robust, but self-limited response to a target antigen. The growing interest over the past two decades in harnessing the immune system to eradicate cancer has been accompanied by heightened efforts to characterize cytokines and exploit their vast signaling networks to develop cancer treatments. The goal of this paper is to review the major cytokines involved in cancer immunotherapy and discuss their basic biology and clinical applications. The paper will also describe new cytokines in pre-clinical development, combinations of biological agents, novel delivery mechanisms, and potential directions for future investigation using cytokines.

## 1. Introduction

Cytokines are molecular messengers that allow the cells of the immune system to communicate with one another to generate a coordinated, robust, but self-limited response to a target antigen. While many forms of communication of the immune system occur through direct cell-cell interaction, the secretion of cytokines enables the rapid propagation of immune signaling in a multifaceted and efficient manner. The growing interest over the past two decades in harnessing the immune system to eradicate cancer has been accompanied by heightened efforts to characterize cytokines and exploit their vast signaling networks to develop cancer treatments.

Cytokines directly stimulate immune effector cells and stromal cells at the tumor site and enhance tumor cell recognition by cytotoxic effector cells. Numerous animal tumor model studies have demonstrated that cytokines have broad anti-tumor activity and this has been translated into a number of cytokine-based approaches for cancer therapy. Recent years have seen a number of cytokines, including GM-CSF, IL-7, IL-12, IL-15, IL-18 and IL-21, enter clinical trials for patients with advanced cancer. There is ongoing pre-clinical work supporting the neutralization of suppressive cytokines, such as IL-10 and TGF-β in promoting anti-tumor immunity. In addition, advances in adoptive cell therapy have relied on the use of cytokines to create an *in vitro,* highly controlled environment for the optimal development of anti-tumor T cells.

To date, two cytokines have achieved FDA approval as single agents for cancer treatment: high-dose, bolus IL-2 for metastatic melanoma and renal cell carcinoma and IFN-α for the adjuvant therapy of Stage III melanoma. The potential of cytokines in the field of cancer immunotherapy is best exemplified by high dose IL-2, which can induce durable complete responses in a subset of metastatic melanoma and renal cell carcinoma patients. However, the extensive pleiotropism and redundancy of cytokine signaling, and the dual function of many cytokines in both immune activation and immune suppression, poses significant challenges to our ability to achieve meaningful anti-tumor responses without also causing treatment-limiting toxicities-a dilemma that is also well exemplified by the low response rates and notorious toxicities of IL-2. Understanding the complex, multifaceted roles cytokines play in the promotion and regulation of the anti-tumor response is critical to the development of effective immunotherapeutic strategies against cancer.

The goal of this review is to survey the major cytokines involved in cancer immunotherapy and discuss their basic biology and clinical applications. The review will also describe new cytokines in pre-clinical development, combinations of biological agents, novel delivery mechanisms, and potential directions for future investigation using cytokines.

## 2. General Features of Cytokines

Cytokines are secreted or membrane-bound proteins that act as mediators of intercellular signaling to regulate homeostasis of the immune system. They are produced by cells of innate and adaptive immunity in response to microbes and tumor antigens. The effects of individual cytokines on immunity depend on several factors, including the local cytokine concentration, the pattern of cytokine receptor expression and the integration of multiple signaling pathways in responding immune cells. The significance of cytokines in tumor immunosurveillance has been demonstrated by the higher frequency of spontaneous cancers seen in mice genetically deficient in type I or II interferon (IFN) receptors or elements of downstream IFN receptor signal transduction [1-3].

Cytokine signaling is characterized by a significant degree of pleiotropism, in which one cytokine has the ability to act on many different cell types to mediate diverse and sometimes opposing effects (Table 1). This has proven to be one of the primary limitations to IL-2 therapy, due to the dual function of IL-2 as a potent activator of the T effector compartment as well as the T regulatory compartment. Another important property of cytokine signaling is its degree of redundancy, in which multiple cytokines have the same functional effects. This redundancy can make the therapeutic manipulation of cytokines somewhat challenging since modification of one cytokine can be compensated by others.

Cytokines play complex and often opposing roles in the development of the immune system, host defense, and tumor immunobiology. Thus, understanding the biological activities and mechanisms of action of these elements is central to developing cytokine-based immunotherapy in cancer treatment.

## 3. Classification of Cytokines and Cytokine Receptors

**Figure 1.**
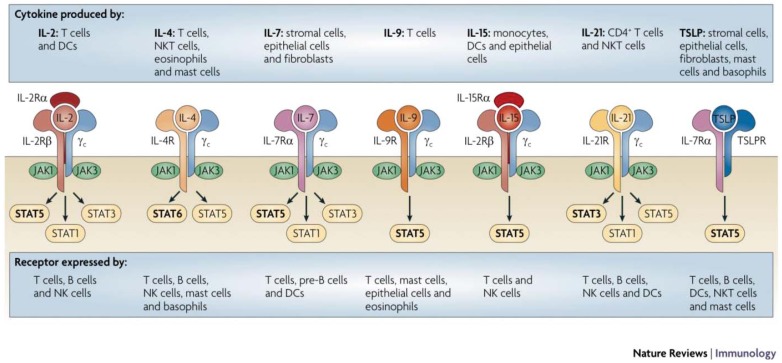
Structural diagram of the major cytokine receptor families. Note that related receptors share common signaling chains and biologic activity is related to the presence of cytokine-binding chains, spatial orientation of the receptor complex and the temporal and cellular pattern of receptor expression. The physiologic impact of receptor signaling is summarized in Table 1. Reprinted by permission from Macmillan Publishers Ltd: Nature Reviews Immunology 2009 [4].

Cytokines signal through a series of common and shared receptors, which have proven useful for a more functional classification of cytokines (Figure 1). To date, there are seven cytokine receptor families (Table 2): Type I cytokine receptors, Type II cytokine receptors, immunoglobulin superfamily receptors, tumor necrosis factor (TNF) receptors, G-protein coupled receptors, transforming growth factor β (TGF-β) and the recently described IL-17 receptors. This chapter will focus on cytokines that signal through the Type I and II cytokine receptor families, as these have the most immediate clinical potential.

### 3.1. Type I Cytokine Receptors

The Type I cytokine receptors, which include receptors for IL-2, IL-4, IL-7, IL-9, IL-15 and IL-21, share a common signaling subunit, the common γ chain (γc), that complexes with a cytokine specific moiety to initiate intracellular signals through the coordinated activity of Janus kinases (JAK) 1 and 3 and signal transducers of activated T (STAT) molecules (Figure 1) [4]. Additional Type I cytokine receptor subgroups include the granulocyte/monocyte colony stimulating factor (GM-CSF) and IL-6 receptor families, which share a common gp130 receptor subunit that mediates complex multi-pathway signal transduction in its target cells [5-8]. The gp130 signal transduction component is utilized by several receptor complexes, including IL-6, IL-11, leukemia inhibitory factor (LIF), oncostatin M, cardiotrophin-1 and ciliary neurotrophic factor, that have redundant and pleiotropic effects on the immune, hematopoietic and nervous systems [9]. Likewise, IL-3, IL-5 and GM-CSF are also recognized by receptors in a separate GM-CSF receptor subfamily which shares a common β chain that complexes with the cytokine-specific α chain [10].

### 3.2. Type II Cytokine Receptors

The effects of IFN-α, IFN-β, IFN-γ and IL-10 are mediated by Type II cytokine receptors, which are composed of a signaling chain and a ligand binding chain. The sequences of the Type II cytokine receptors resemble tandem Ig-like domains and the intracellular segments are typically associated with a tyrosine kinase of the Janus kinase (JAK) family [11].

### 3.3. Immunoglobulin Superfamily Receptors

The immunoglobulin superfamily receptors contain extracellular immunoglobulin domains and include the receptors for IL-1, IL-18, stem cell factor and monocyte colony stimulating factor [12].

## 4. Current Cytokines in Immunotherapy

In this section, an effort is made to include cytokines that have already advanced into clinical use or have a strong preclinical basis for demonstrating therapeutic benefit in cancer patients.

### 4.1. The Interferons (IFN)

The IFNs are classified by their ability to bind to specific receptors termed Type I, Type II, and the recently-described Type III IFN receptors [13].

#### 4.1.1. Type I Interferons

Type I IFNs, which include IFN-α and IFN-β, have emerged as the most clinically useful IFNs for the treatment of cancer. They are secreted by nearly every cell in the body and are predominantly involved in cellular immune responses against viral infections [14-16]. Type I IFNs induce expression of major histocompatibility complex (MHC) class I molecules on tumor cells and mediate the maturation of a subset of dendritic cells (DC) [17-20]. They can also activate cytotoxic T lymphocytes (CTLs), natural killer (NK) cells and macrophages [21,22]. In addition to their immunologic effects, the Type I IFNs can exert cytostatic and possibly apoptotic effects on tumor cells as well as anti-angiogenic effects on tumor neovasculature [23,24]. Mice with a targeted deletion of the Type I IFN receptor have a higher rate of carcinogen-induced cancer and increased tumor growth in transplantable tumor models, supporting the hypothesis that the type I IFNs are important in tumor immunosurveillance [25,26].

The Type I IFNs all share the same receptor complex (INF-αR1 and INF-αR2). IFN-α and IFN-β are actually families in themselves that comprise over 20 distinct molecules classified according to their ability to activate Type I IFN receptors [14-16,27]. IFN-α is comprised of a group of at least twelve distinct proteins. Recombinant IFNα-2a, IFNα-2b and IFNα-2c differ by one or two amino acids and are the isoforms most commonly used in the clinic [28]. Since IFN-α and IFN-β signal through the same receptor, they would be expected to have similar biologic effects and have overlapping indications. This prediction has not, however, been confirmed clinically and the mechanism of anti-tumor activity *in vivo* is not completely defined for this group of IFNs.

IFNs activate the JAK-STAT signaling pathway. IFN-α and IFN-β stimulate the activity of JAK1 and TYK2 proteins, leading to STAT1 and STAT2 tyrosine phosphorylation, and ultimately induce IL-4 secretion and subsequent activation of B cells [25,26,29,30]. IFN-α also causes direct apoptosis of tumor cells in a caspase-dependent manner, which may contribute to the well-known properties of type I and II IFNs to enhance tumor cell antigen expression as well as co-stimulatory and co-inhibitory receptors that are essential to the type of immune reaction resulting between tumor and effector cells [31]. At low doses, IFN-α acts as an anti-angiogenic agent [32]. While the specific mechanisms of IFN-mediated tumor rejection in animal models have not been fully elucidated, IFN-α has been the most widely investigated cytokine for human cancer treatment and may prove to be a valuable component of combinatorial strategies for immunotherapy of solid tumors.

##### 4.1.1.1. Clinical Applications of IFN-α

IFN-α is the only currently approved adjuvant therapy for patients with high-risk Stage II or Stage III melanoma based on a series of cooperative group, multi-institutional clinical trials. While these trials initially showed improvement in both relapse-free survival and overall survival, subsequent long term follow-up data led to the finding that with longer follow-up time, the tails of the curves for relapse-free and particularly overall survival no longer demonstrated significant benefits for IFN-α over the comparators [33-35]. In a more recent meta-analysis of 14 randomized clinical trials enrolling 8,122 patients over an 18-year period, IFN-α was associated with a significant improvement in disease-free survival in 10 of 17 comparisons and improved overall survival in four of 14 comparisons [36]. These authors concluded that IFN-α should remain the standard for adjuvant treatment in high risk melanoma patients. Further support and mechanistic insight was provided by a report from the Hellenic Oncology Group demonstrating a strong association between favorable outcomes and the development of one or more autoimmune phenomena (vitiligo or thyroid abnormalities, or serologic evidence of autoimmune phenomena) among high-risk melanoma patients during or after adjuvant IFN-α [37], although subsequent analyses from US and European studies did not find this association [38]. Other regimens of IFN-α adjuvant therapy for melanoma have been associated with improved relapse-free survival or distant relapse-free survival but no overall survival benefit. While high-dose IFN-α is the most commonly-used regimen when adjuvant IFN-α is offered to melanoma patients in the US, there is a great need for therapies with a better therapeutic index, and a variety of other regimens are currently under investigation in this setting. The lack of an IFN-α-containing “control” arm in some large studies but inclusion in others is further evidence of existing controversy over the true benefit of this adjuvant therapy.

In addition to the treatment of melanoma, IFN-α is approved for the treatment of some hematologic malignancies, AIDS-related Kaposi's sarcoma, and as a component in an anti-angiogenic combination regimen with bevacizumab for advanced renal cancer. IFN-α has been particularly effective as therapy for hairy cell leukemia (HCL) and chronic myelogenous leukemia (CML). For treatment of HCL, IFNα-2b given at a low dose of 2 million units/m^2^ subcutaneously three times a week for one year resulted in an overall response rate of 77% with a complete response rate of 5%, and this low dose was well-tolerated. Patients with an intact spleen appeared to achieve an even greater complete response rate of 25–35% in follow-up studies, suggesting the importance of early initiation of cytokine therapy in this disease. While relapses are common in HCL following IFN-α therapy, retreatment provides remissions in most patients [39,40]. Nevertheless, the advent of nucleoside analogs, with a complete response rate close to 90% and durable remissions in most patients, has relegated IFN therapy to second-line treatment in patients with refractory disease or in those with contraindications to nucleoside analog drugs [41].

While the complex mechanisms of action of IFN-α are not fully understood, one well-established function is its ability to enhance tumor antigen recognition by increasing MHC class I expression. The particular importance of this action is suggested by an intriguing parallel recently discovered between HCL and melanoma: they both carry a mutation in the BRAF gene [42]. In melanoma, BRAF mutations appear to enable immune evasion by suppressing the expression of melanoma differentiation antigens that typically serve as the targets for anti-tumor T cells [43]. This antigen expression can be restored by BRAF inhibitors. If the BRAF mutation is also found to cause a down regulation of tumor antigens in HCL, this would provide a compelling explanation for why two seemingly disparate diseases are among the rare responders to the actions of IFN-α. If the increase in tumor antigen expression proves to be the primary therapeutic mechanism of IFN-α, then this would also provide rationale for further investigation into IFN-α in combination with BRAF inhibitors.

##### 4.1.1.2. Toxicity of IFN-α

Experience with IFN-α administration has resulted in established guidelines for recognition and management of toxicities and side effects. The toxicity profile of IFN-α is usually dose-related, and most side effects can be managed without discontinuation of treatment. Constitutional symptoms including fever, fatigue, headaches, gastrointestinal symptoms and myalgias are quite common and will likely occur in 80% or more of patients. IFN-α also produces increases in hepatic enzymes in some patients, particularly during the high-dose intravenous period when patients should be monitored frequently and therapy held or dose-reduced for those with hepatic enzyme elevations during therapy. Thrombocytopenia, leukopenia and neutropenia are common and can also be readily managed with dose reductions, or rarely, transfusions [44,45]. More serious are the neuropsychiatric issues, which include depression (45%), confusion (10%), and mania (<1%) [44-46]. In some studies of IFN-α depression was highly significant and rare suicides were reported [47]. Permanent alterations of the immune system have also been reported, including common development of vitiligo and hypothyroidism and rare occurrence of sarcoidosis, lupus, rheumatoid arthritis, polymyalgia rheumatica and psoriasis [48].

In view of these observations and those detailed above, the mechanisms of IFN-α in melanoma and the possible association with selected parameters of altered immune control suggest that it may be possible to identify underlying tumor or patient factors predictive of benefit prior to initiating therapy and to avoid the toxicities of IFN-α in those predicted to have no benefit. To date, attempts to find such predictors among the polymorphisms in the HLA system and single nucleotide polymorphisms of the checkpoint protein CTLA-4 have not been fruitful.

##### 4.1.1.3. Clinical Potential of IFN-β

IFN-β is produced not only by leukocytes but also by some tumors [49]; nevertheless, its therapeutic potential in immunomodulatory strategies has been demonstrated both for suppression of autoimmune reactivity (four different formulations of the drug are approved for multiple sclerosis) as well as for immunostimulation in the treatment of malignancy in a number of preclinical models. In comparative analyses of the antitumor effects of the Type-I IFNs, IFN-β is more potent than IFN-α in inducing antiproliferative effects in preclinical cancer models [50-52]. In spite of its higher antiproliferative potential compared to IFN-α, the clinical use of IFN-β in cancer therapy has been limited by its low bioavailability and sustained side effects; however, this may be overcome by delivery in different routes and schedules.

#### 4.1.2. Type II Interferons

The only member of this family is IFN-γ, which binds to a distinct receptor complex (IFNγ R1 and IFNγ R2) [27]. The Type II IFN receptor is a subset of the type II cytokine receptors [53,54]. IFN-γ is secreted by NK cells, NKT cells, Th1 CD4+ T-cells, CD8+ T cells, antigen presenting cells (APCs) and B-cells [55,56]. IFN-γ activates macrophages and induces the expression of MHC class I, MHC class II and co-stimulatory molecules on APCs [57]. Additionally, IFN-γ induces changes in the proteasome leading to enhanced antigen presentation [58,59]. IFN-γ also promotes Th1 differentiation of CD4+ T cells and blocks IL-4 dependent isotype switching in B cells [57,60]. IFN-γ activates the JAK-STAT signaling pathway by phosphorylating JAK1 and JAK2 proteins which produces a recruitment site for STAT1.

Clinical Applications of IFN-γ. Numerous murine studies originally suggested an important role for IFN-γ in tumor immunity: mice with targeted deletion of IFNγ or the Type II IFN receptor have an increased risk of spontaneous and chemically-induced tumors compared to controls [1,3,26,61]. IFN-γ is cytotoxic to some malignant cells and has modest anti-angiogenic activity [62-64]. IFN-γ may also be an important regulator of anti-tumor activity mediated by other cytokines, in particular IL-12 and probably IL-2 [65,66]. However, IFN-γ has demonstrated very limited clinical utility in cancer therapy, which may in part be due to its immune regulatory role in the activation of myeloid derived suppressor cells and a narrow therapeutic index. While the clinical potential for IFN-γ has been limited, its secretion or the expression of its gene in effector lymphocytes is used commonly as a readout in laboratory assays for antigen-specific effector cell function.

#### 4.1.3. Type III Interferons

This recently-discovered family consists of IFN- γ1, IFN- γ2, and IFN- γ3 which activate an IL-10 receptor 2 subunit and IL-28 receptor α subunit complex [67]. This may prove to be an important subgroup in the future, but at this time, the therapeutic uses for Type III IFNs have not yet been established. Interestingly, while type III IFNs share many of the same biological activities as the type I IFNs due to a similar signaling pathway, type III IFN receptor expression is mostly restricted to cells of epithelial origin (e.g., liver, lung) and notably absent from hematopoietic cells and the central nervous system. This observation has inspired ongoing efforts to determine whether type III IFNs will be able to exert the antitumor effects of IFN-α without the associated hematologic and neurologic toxicities [68].

### 4.2. Interleukin-2

#### 4.2.1. Biology and immunology

Interleukin-2 (IL-2), as well as other members of the IL-2-related family of T cell growth factors (e.g., IL-4, IL-7, IL-9, IL-15, and IL-21), utilize a common receptor signaling system that results in the activation and expansion of CD4+ and CD8+ T cells.

The biological effects of IL-2, a 15.5 kDa variably glycosylated protein comprised of four antiparallel α-helices, are mediated by the IL-2 receptor, a trimeric complex composed of α (CD25), β (CD122) and γ (CD132) chains. The β and γ chain are involved in signaling, while the ligand-specific α chain is only involved in cytokine binding. These subunits form a high (αβγ), intermediate (βγ) or low affinity receptor (α) depending on which of the chains are in the cell surface complex [69,70]. Although the β and γ chains are expressed on T cells, B cells and NK cells [71], the α chain is inducible and is expressed only by T cells but is present on several phenotypically and functionally distinct classes of T lymphocytes. The predominant cellular source of IL-2 is the CD4 T cell, specifically the Th1 subset, and its major physiologic role of IL-2 is to promote the activation and proliferation of T and NK cells in an autocrine and paracrine manner [72]. In contrast to T cells, NK cells express the intermediate affinity IL-2 receptor (no α subunit). Exposure of NK cells to IL-2 results in proliferation, enhanced cytolytic activity and secretion of other cytokines. B cells also express intermediate affinity IL-2 receptors and can secrete IL-2 in cooperation with other cytokines, resulting in B cell proliferation and differentiation [71].

IL-2 also plays a major role in suppressing T cell responses. A subpopulation of CD4+ T cells, characterized by high levels of CD25 and the forkhead/winged helix transcription factor FoxP3, function to suppress self reactive T-cells. These regulatory T cells (Tregs) maintain tolerance and prevent autoimmunity after activation of effector T-cell responses [73]. In murine models, depletion of CD4+FoxP3+ Tregs enhances tumor rejection and improves therapeutic responses to cancer vaccines by promoting the function of CD8+ cytotoxic T lymphocytes [74]. The mechanisms by which Tregs inhibit the function of CD8+ CTLs are incompletely understood. However, recent *in vivo* studies show competition for IL-2 is a critical pathway by which Tregs limit CD8+ T cell expansion and effector differentiation [75]. Furthermore, the loss of IL-2 signaling, as demonstrated in mice with a targeted deletion of IL-2 or the IL-2 receptor, leads to a generalized inflammatory syndrome and an often fatal autoimmune colitis [74,76-78], providing additional evidence of the role of IL-2 not only as an activator of immune responses but also as a key mediator of immune tolerance. This relatively recent insight into IL-2 as a regulatory cytokine, rather than a purely stimulatory T cell growth factor, suggests that the use of IL-2 in the clinical setting needs to be re-evaluated. An important area of further investigation will be a more careful analysis of the dosing, schedule and kinetics of IL-2 administration on specific T cell subsets.

#### 4.2.2. Clinical Applications of IL-2

IL-2 plays a pivotal role in the treatment of patients with metastatic melanoma and renal cell carcinoma. Malignant melanoma is a tumor of melanocytes and many primary cutaneous melanomas exhibit histologic regression and infiltration of T and NK cells at the time of clinical detection [79]. In general, melanoma has not been responsive to cytotoxic chemotherapy. Thus, early studies focused on the generation of effective immune responses in melanoma patients. Initial work in the Surgery Branch of the National Cancer Institute found that adoptively transferred IL-2-activated peripheral blood mononuclear cells with the phenotype and functional characteristics of activated NK cells, supported with concomitant administration of IL-2 in high doses, resulted in significant tumor regression in patients selected for normal organ function and good performance status. Further investigation of these encouraging results suggested that therapeutic benefit could be seen in a subset of patients treated with high doses of IL-2 alone.

High-dose IL-2 induces objective clinical responses in 15–20% of patients with advanced melanoma and durable complete responses in 5–7% of these patients [80,81]. In order to reduce the IL-2-related side effects, a variety of modifications to the high-dose IL-2 regimens have been tested in patients with melanoma, including alterations of dose, schedule and route, as well as chemical alterations of IL-2 molecular structure that alter its cellular targets. Other modifications, including the addition of toxicity modulators such as drugs with anti-inflammatory properties or anti-angiogenic agents, have also been tested. Unfortunately, none of these modifications has led to improved therapeutic index, and current strategies are revisiting the concept of investigating host and tumor biologic and immunologic properties that might predict benefit and allow for selection of patients with improved therapeutic index. Additional efforts are underway to develop combinations of immunomodulators that combine cytokine with other agents to enhance antitumor immunity. IL-2 continues to play an important role as a T cell growth factor in adoptive T cell therapies (as a component of the T cell expansion procedure and administered following cell infusions) but is likely to be supplanted or augmented by other cytokines such as IL-15, IL-21 and IL-7 as their advantages and possible synergies become better understood.

Metastatic renal cancer, particularly the clear-cell histology that comprises the majority of cases, is another tumor that is inherently resistant to cytotoxic agents and has shown responsiveness to immune modulators such as IFN-α and IL-2. The response rate with high-dose intravenous bolus IL-2 is around 25% for metastatic renal cell carcinoma patients, similar to that seen in melanoma patients and with a similar rate of durable complete response in the 7% range. The overall clinical responses with high-dose IL-2 have been relatively durable, with median response durations of 24–54 months and with over 80% of complete responders being long-term survivors [82]. Thus, high-dose IL-2 should remain in the armamentarium of the experienced clinical oncologist for advanced renal cell carcinoma. Interactions and optimal sequencing of IL-2 with tyrosine kinase inhibitors, now widely used for the frontline and subsequent treatment of advanced renal cancer, need to be better understood in order to provide the optimal therapy for all patients with this disease.

#### 4.2.3. Toxicities of IL-2

The toxicity profile of IL-2 is largely associated with a capillary leak syndrome, which is characterized by hypotension, tachycardia and peripheral edema secondary to third space fluid accumulation. In addition, IL-2 can cause constitutional symptoms such as fever, chill and fatigue, gastrointestinal side effects such as nausea, vomiting, anorexia, transaminase elevation, cholestasis and diarrhea [83]. In addition to hypotension, IL-2 may also induce pulmonary edema, cardiac arrhythmias, myocarditis, reversible renal and hepatic dysfunction, pruritus, electrolyte abnormalities, thrombocytopenia, anemia and coagulopathy. Rarely IL-2 may also induce confusion, disorientation or visual hallucinations. Although early studies with IL-2 reported a 2% mortality rate, generally related to gram-positive sepsis, current IL-2 centers that routinely use prophylactic antibiotics report no mortality [84-86]. In experienced centers, IL-2-related toxicity can usually be easily managed and all side effects are reversible upon cessation of treatment.

#### 4.2.4. Predictive Biomarkers of IL-2

There has been intense interest in the discovery of predictive biomarkers for better selection of patients likely to respond to IL-2 therapy for both RCC and melanoma. A defined polymorphism in the CCR5 gene (CCR5Δ32) was associated with decreased survival following IL-2 administration in patients with Stage IV melanoma compared to patients not carrying the deletion [87]. Increased pre-treatment serum vascular endothelial growth factor (VEGF) and fibronectin levels were associated with a poor response to IL-2 and a decreased overall survival [88]. Preliminary studies suggested that elevated levels of carbonic anhydrase IX (CAIX) in renal cell carcinoma patients conferred a better response to IL-2 therapy compared to patients with tumors demonstrating normal or low levels [89,90], but larger trials that included additional characteristics raised the possibility of more discriminating markers that remain under active investigation. Other studies have focused on assessing the number, phenotypic characteristics, and functional status of CD4+FoxP3+ Tregs in melanoma and renal cell carcinoma patients undergoing standard high-dose IL-2 administration. While the number of Tregs increased after exposure to IL-2 and remained elevated in patients with disease progression, patients who responded to IL-2 demonstrated a decrease in Tregs to normal levels within four weeks of completing IL-2 treatment. Ultimately, it will be essential to identify predictive factors (specific for the intervention and not simply prognosticators for the natural history of the disease) in sufficient numbers to be validated in large patient cohorts.

### 4.3. IL-2 Related Cytokines

#### 4.3.1. Interleukin 7

IL-7, a member of the small four α-helix bundle family of cytokines, is one of the IL-2-related cytokines that signal through the γc receptor subunit to exert influence over T cell survival, proliferation and homeostasis. The critical role of IL-7 in T cell development is evidenced by the finding that IL-7 receptor mutations lead to an absence of T cells and the development of severe combined immunodeficiency (SCID) [91]. IL-7 is a homeostatic cytokine and functions as a limiting resource that provides continuous signal to resting naïve and memory T cells [92]. During conditions of lymphopenia, IL-7 then accumulates which leads to an increase in both T cell proliferation and T cell repertoire diversity. IL-7 also plays a role in B cell development and its receptor is found on immature B cell progenitors. A potential therapeutic advantage of IL-7 over IL-2 is its selectivity for expanding CD8+ T cell populations over CD4+FOXP3+ regulatory T cells [93]. In murine models, recombinant IL-7 has been found to augment antigen-specific T cell responses after vaccination and adoptive cell therapy [94,95], and this is now being evaluated in humans through two clinical trials [96]. Another important area of investigation is the potential role of IL-7 in promoting T-cell recovery after chemotherapy or hematopoietic stem cell transplantation. Early phase clinical trials on patients with advanced malignancy have demonstrated recombinant IL-7 to be well-tolerated with limited toxicity at biologically active doses (in which the numbers of circulating CD4+ and CD8+ T cells increased by 3–4 fold), suggestive of a broad therapeutic index.

#### 4.3.2. Interleukin 15

**Figure 2.**
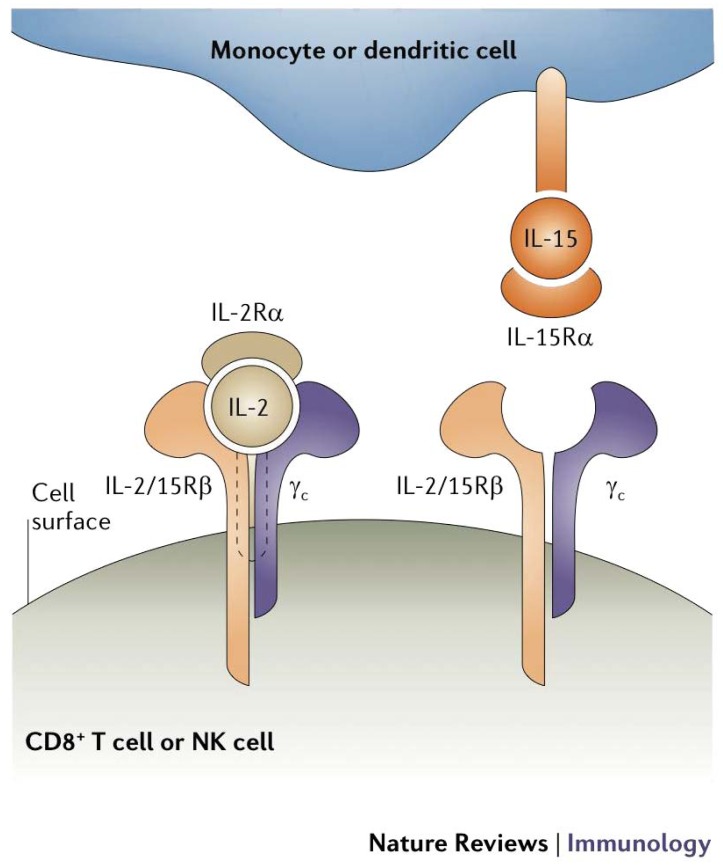
Structural differences in IL-2 and IL-15 signaling. IL-2 and IL-15 bind two common subunits (IL-2R/15Rβ and γ_c_) which signal through the same JAK and STAT molecules, accounting for the similarity in their actions. However, while IL-2 is a secreted cytokine and binds receptors on the surface of activated cells, IL-15 is primarily membrane bound and must be presented through cell-cell contact, which then involves additional costimulatory signals that further modify the cell response. Reprinted by permission from Macmillan Publishers Ltd: Nature Reviews Immunology, 2006 [104].

IL-15 is also one of several cytokines structurally similar to IL-2 that signal through the γc receptor subunit and have recently entered clinical investigation for cancer and hematologic malignancies [97]. While both IL-2 and IL-15 provide early stimulation for T cell proliferation and activation, IL-15 acts to block IL-2-induced apoptosis [98-100]. IL-15 also supports the persistence of memory CD8+ T cells, which may be important for maintaining long-term anti-tumor immunity [101]. IL-15 has demonstrated significant therapeutic activity in several pre-clinical murine models of cancer [102]. These effects are mediated through direct activation of CD8+ effector T cells in an antigen-independent manner [103]. Importantly, IL-15 must be presented in reverse orientation (Figure 2) to CD8+ T cells by nearby bone marrow (BM)-derived cells on the high affinity IL-15Rα, which may account for some of its differences in action from IL-2 despite shared receptor subunits [104]. IL-15 has just entered Phase I trials in human subjects and is expected to have value in a variety of immunotherapeutic strategies, including both *in vivo* and in *ex vivo* strategies for adoptive cell therapies.

#### 4.3.3. Interleukin 21

IL-21 is a type I cytokine with close homology to IL-2, IL-4, and IL-15 that shares with these cytokines plus IL-7 and IL-9 the γc receptor subunit and a cytokine-specific α-receptor. IL-21 signaling is distinguished from that of other γc cytokines by activating primarily STAT1 and STAT3 [105]. Produced primarily by activated CD4+ T cells, IL-21 has pleiotropic effects, including the promotion of CD4+ and CD8+ T cell proliferation and enhancement of CD8+ T cell and NK cell cytotoxicity without promoting activation-induced cell death [106-108]. Although the role of IL-21 in Th1/Th2 differentiation is unclear, it is required for normal humoral responses and believed to influence the transition from innate to adaptive immunity [109]. IL-21 has demonstrated therapeutic activity in murine tumor models of melanoma and has recently entered Phase I clinical trials with modest preliminary results [110-113]. Like the other γc cytokines (IL-2, IL-7, IL-15), IL-21 has recently started playing a role in adoptive T cell strategies based on its ability to enhance significantly the *ex vivo* generation and affinity of antigen-specific T cells [114].

### 4.4. Additional Cytokines with Clinical Application

#### 4.4.1. Interleukin-6

IL-6 is a type I cytokine normally produced by macrophages, endothelial cells and some activated T cells. It signals through a receptor via a signal transducing subunit gp130, which is shared in common with a subfamily of cytokines, including IL-11 and IL-27 [115]. Among its diverse functions, IL-6 mediates inflammation and plays a critical role in hematopoiesis and stem cell amplification and differentiation [116]. Overexpression of IL-6 appears to play a role in the pathogenesis of many cancers, including breast, renal cell, colon, prostate, bladder, in addition to B-cell malignancies, most notably myeloma. Additionally, elevated serum levels of IL-6 have been shown to carry a poorer prognosis in all these tumor types. Some cancer cells, such as myeloma, begin to secrete high amounts of IL-6 to serve as an autocrine growth factor that promotes their own survival. In fact, one of the reasons corticosteroids are effective agents against myeloma is likely because they inhibit IL-6.

Our understanding of IL-6 as a protumorigenic cytokine has led to therapeutic strategies using monoclonal antibodies to IL-6 and the IL-6 receptor as well as IL-6-conjugated toxins, although a limitation to this approach is the wide expression of IL-6R by many normal cells [116]. There have been a few early phase clinical trials using monoclonal antibodies to IL-6, with mixed results. In one of the largest studies, 12 patients with refractory myeloma were treated with a chimeric human-mouse IL-6 antibody and 11 patients had stabilization of disease but none achieved a clinically significant response (defined as greater than 50% reduction in M protein) [117]. However, there may be a more promising role for anti-IL-6 therapy in the management of cancer-related, inflammation-associated symptoms such as cachexia, fever, and pain [118].

#### 4.4.2. Interleukin-12

IL-12 is a heterodimeric cytokine containing a 35 kD and a 40 kD subunit that signals through a receptor of the Type I family of cytokine receptors. IL-12 is produced mainly by phagocytic cells in response to antigenic stimulation, leading to cytokine production, primarily of IFN-γ, from NK and T cells [119]. IL-12 also acts as a growth factor for activated NK and T cells, promotes CD4+ T cell differentiation into Th1 CD4+ T cells and enhances the activity of CD8+CTLs [120-122]. In addition, IL-12 elicits anti-angiogenic effects that require IFNγ and are mediated by IFNγ-inducible protein 10 (IP-10), a chemokine induced in a variety of cells in response to IFN-γ and lipopolysaccharide [123].

IL-12 has demonstrated anti-tumor activity in murine models of melanoma, colon carcinoma, mammary carcinoma and sarcoma [63,124-129]. Experimental investigation of the mechanism of IL-12 activity using mice with molecularly targeted defects suggests that the effector cells involved in the antitumor immune response to IL-12 differ by species, by tumor model and by dose and schedule of IL-12 as well as other cytokines and elements of the immune microenvironment. For example, in the B16 murine melanoma model, a significant role for NK cells has been demonstrated in mediating anti-tumor immunity with high doses of IL-12, while anti-tumor responses at low doses of IL-12 appear to be mediated by NKT cells [130].

IL-12 has been studied as a therapeutic cytokine for cancer in extensive preclinical investigations and a variety of clinical settings. IL-12 also exhibits anti-angiogenic effects mediated by IFNs, particularly IFN-γ, and IP-10 [130]. Based on these provocative pre-clinical studies, IL-12 was evaluated in patients with metastatic melanoma. In a Phase I clinical trial, the objective response rate was less than 5%. Although the response rates were low, patients who did respond had sustained serum levels of IFN-γ, IL-15 and IL-18, suggesting that sustained IFN-γ production might result in better responses. In a phase I trial combining IL-12 with low doses of IL-2, sustained levels of IFN-γ and expansion of NK cells were observed, although only 1 patient achieved a partial response [131]. IL-12 appears to have the potent ability to induce counterregulatory cytokines such as IL-10 that may abrogate its immunostimulatory properties, depending on the dose and schedule of administration, but it may have promise as a component of the immune adjuvant in certain tumor vaccine strategies as well as in locoregional delivery vehicles such as plasmid electroporation [131,132].

#### 4.4.3. Interleukin-18

IL-18, a 24 kDa, non-glycosylated polypeptide, was initially identified as IFN-γ inducing factor and is structurally related to IL-1 [133-135]. IL-18 stimulates IFN-γ secretion by NK and CD8+ T cells and enhances their cytotoxicity [134-136]. Other functions of IL-18 include macrophage activation, development of Th1 helper CD4+ T cells, increased expression of FasL on lymphocytes, and promotion of angiogenesis [137,138]. Phase I clinical trials documented the safety of IL-18 and found increased levels of serum IFN-γ and GM-CSF in patients after receiving intravenous IL-18. The clinical responses have been modest with only two objective responses in 26 patients in one trial and three stable disease patients in another study [139]. Additional investigation will be required to discover a niche for IL-18 in cancer immunotherapy strategies.

#### 4.4.4. Granulocyte-Macrophage Colony Stimulating Factor

GM-CSF, a heterogeneously glycosylated 14 to 35 kd polypeptide, was initially identified as a mediator of hematopoiesis and monocyte-macrophage differentiation [140]. GM-CSF is also a highly pleiotropic cytokine and is closely related to IL-3, which stimulates multilineage myelopoiesis, and IL-5, the predominant growth factor for eosinophil, by way of a common β chain on the GM-CSF receptor [141]. The receptors for GM-CSF, like those for IL-3 and IL-5, are composed of two subunits, a ligand-specific α chain and a common β chain. GM-CSF is produced by monocytic cells and T cells and promotes the maturation of dendritic cells. The potential for GM-CSF to stimulate immune responses has been shown in many tumor models, including a murine melanoma in which transgenic expression of GM-CSF provided protection to subsequent tumor challenge in over 90% of the animals [142], and promising results have been observed in other tumors when used alone and in combination with other immunomodulators such as checkpoint-blocking antibodies [143,144]. The anti-tumor activity of GM-CSF appears to be related to its ability to activate macrophages and dendritic cells [145,146]. GM-CSF also matures DCs leading to upregulation of co-stimulatory molecules and CD1d receptors, involved in antigen presentation [147]. Initial studies suggested that CD4+ and CD8+ T cells mediated GM-CSF-stimulated antitumor immunity, but recent models using CD1d deficient mice support a critical role for NKT cells in GM-CSF anti-tumor immune responses. More recently, it has been suggested that GM-CSF may serve a more regulatory role in the induction of DC-mediated T cell immunity through complex interactions with milk fat globule-8 (MFG-8), a glycoprotein on antigen-presenting cells that contributes to the control, under various biologic conditions, of immunologic responses resulting from their interactions with T cell subsets [148].

Recombinant GM-CSF was approved by the FDA to shorten the time to neutrophil recovery and reduce the incidence of infections following induction chemotherapy in patients with acute myelogenous leukemia. GM-CSF is also used to mobilize hematopoietic progenitor cells into the peripheral blood for leukapheresis collection and enhance engraftment and myeloid reconstitution after autologous and allogeneic bone marrow transplantation. The substantial preclinical work demonstrating a variety of immunostimulatory properties for this molecule, in particular through its transgenic expression in tumor cells to create a promising tumor vaccine [149], has led to extensive testing in a variety of immunotherapeutic strategies. Single agent GM-CSF has been reported to have antitumor activity in melanoma when injected directly into metastatic lesions [150].

## 5. Role of Cytokines in Immune Suppression

Cytokines are responsible for the induction of active immune responses against tumors as well as the negative regulation of immune responses in maintaining homeostasis and self-tolerance. Self-tolerance is mediated by two major classes of CD4+FoxP3+ Tregs, and understanding how cytokines regulate the generation and maintenance of Tregs-and how to break this component of tolerance to achieve and maintain successful antitumor immunity—is an important area of current investigation [151]. An IL-10-dependent Type I T regulatory (Tr1) cell arises in the periphery upon encountering antigen in a tolerogenic environment and mediates immune suppression. In contrast, a naturally occurring CD4+FOXP3+ T cell population (nTreg) mediates immune suppression in a contact-dependent, cytokine-independent and antigen non-specific manner [152]. Throughout the process cytokines regulate the number and functionality of these cells as well as the effector cells that fight pathogens and tumors. It is this delicate balance between effector and regulatory T cells that is critical for influencing the rejection or progression of tumors. IL-2, transforming growth factor-β and IL-10—and probably other cytokines—have been shown to modulate the generation of Tregs and may be involved in the fine balance between effector and regulatory populations [153,154].

Another important player in the regulation of the immune system are myeloid-derived suppressor cells (MDSC), which expand during cancer, inflammation and infection [155]. This is a heterogeneous group of cells that are described to be potent suppressors of T-cell response. The activation of MDSCs has been attributed to IFN-γ, IL-4, IL-13, and TGF-beta, while expansion of MDSCs is promoted by many other factors, including GM-CSF, M-CSF, IL-6, and VEGF.

### 5.1. Interleukin-10

Interleukin-10 (IL-10) terminates immune responses through its inhibitory actions on macrophages and dendritic cells. IL-10 inhibits the production of IL-12 by activated macrophages and dendritic cells and also decreases their expression of class II MHC molecules. IL-10 is a homodimeric 17–20 kDa glycoprotein with an α-helical tertiary structure that signals through a JAK-STAT complex, the specific components of which vary with the target cell type. The IL-10 receptor is a member of the IFN receptor family and has two subunits, an α subunit that is primarily expressed on immune cells, with the highest density on monocytes and macrophages, and a β subunit that is found ubiquitously. IL-10 is produced by many different cells of the immune system, including T and B lymphocytes, monocytes, dendritic cells, and NK cells [156]. While IL-10 generally functions as an immunosuppressive cytokine, polarizing T cell responses towards the Th2 phenotype associated with other suppressive cytokines like IL4, IL13, and TGFβ, IL-10 can also have stimulatory effects in certain circumstances, including the stimulation of macrophage phagocytosis and NK cytotoxicity while suppressing inflammatory cytokines, antigen-presentation and T cell response [157,158]. IL-10 can act as a growth factor for malignant B cells such as the plasma cell clone of myeloma and other B cell lymphoproliferative diseases [159]. There are also pre-clinical data suggesting that one mechanism of anti-tumor activity induced by CTLA-4 blockade may be through a decrease in IL-10 secretion [160].

Various tumor cells have been shown to produce IL-10, including cells from non-small cell lung cancers, melanomas, gliomas, leukemias, and lymphomas [161-165]. Furthermore, increased IL-10 production has been observed in tumor-infiltrating lymphocytes from patients with aggressive malignancies such as advanced non-small cell lung cancer and in peritoneal monocytes from patients with malignant ascites from advanced ovarian cancer [166,167]. Constitutional IL-10 promoter polymorphisms have been associated with the susceptibility to certain malignancies, suggesting that this cytokine may play a critical role in some aspect of tumor immunosurveillance [168,169].

## 6. Additional Cytokines with Uncertain Application

An area of recent investigation has been the IL-17/IL-23 axis surrounding TH17cells, a subset of helper T cells known to have an important pro-inflammatory role in autoimmune disease and potentially in tumor immunity as well [170]. IL-23 is produced by dendritic cells and macrophages and promotes the differentiation and expansion of TH17 cells, which then produce IL-17 (in addition to other cytokines including IL-2, GM-CSF, IFN-γ, and TNF.) The role of TH17cells and IL-17 remains controversial; TH17cells have been found in human and mouse tumors and are thought to play a role in the tumor microenvironment, but whether this role is predominantly pro- or anti-tumorigenic is unclear [170,171]. IL-17 was found to promote tumor vascularization and growth in immunodeficient mice, but was separately found to mediate an antitumor effect in immunocompetent mice [172,173]. IL-23 has also demonstrated both pro-tumor and anti-tumor effects in different mouse models: IL-23 deficient mice have a decreased incidence of chemically-induced tumors, while increasing IL-23 at the tumor site or systemic IL-23 leads to tumor inhibition and increased survival [174-176].

Along similar lines, TGF-β, which is abundantly secreted by tumor cells and surrounding stromal cells, has been found to promote invasion and metastases for many human cancers [177,178]. Various methods of TGF-B inhibition or blockade have conferred decreased bone metastases, decreased tumor burden and increased survival in animal models. However, TGF-B also functions as a tumor suppressor in premalignant cells, and inhibits cell growth, induces apoptosis, suppresses growth factors such as the proto-oncogene c-myc [179].

The dual functions of IL-17, IL-23, and TGF-beta as tumor promoters and tumor suppressors has generated controversy surrounding their potential as targets for cancer immunotherapy. Further characterization of their multifaced roles in different contexts and environments will be essential in determining their therapeutic value.

## 7. Novel Strategies for Cytokine Delivery

A variety of innovative strategies for delivery of therapeutic cytokines have been promising in treatment of malignancy. These include cytokine-antibody fusion molecules (immunocytokines), recombinant viral vectors to deliver cytokine genes, transgenic expression of cytokines in whole tumor cells, and chemical conjugation to polyethylene glycol (PEGylation) to improve the kinetics of the cytokine.

### 7.1. Cytokine-Antibody Fusion Molecules

A cytokine-antibody fusion molecule is a genetically engineered fusion protein consisting of an antibody with a functional cytokine and an antigen-binding site designed to deliver cytokines to the tumor microenvironment. The prototype fusion molecule has utilized various antigen-binding moieties fused to recombinant human IL-2 [180]. The therapeutic potential of this approach has been demonstrated using a fusion construct encoding the anti-GD2 ganglioside binding site and IL-2 against a human neuroblastoma tumor in a SCID mouse model. In this system local IL-2 delivery through the fusion molecule resulted in enhanced effector T cell responses and increased tumor cell lysis compared to systemic IL-2 delivery. The fusion molecule was also more proficient than equivalent doses of rhIL-2 in prolonging survival and, in another study, supported proliferation of lymphokine-activated killer cells. Treatment resulted in the accumulation of the fusion molecule in the tumor, which slowed tumor growth and induced a significant immune response. This effect was more pronounced when the bifunctional molecule was injected directly into the tumor, highlighting the importance of local delivery [181,182]. Phase I and II clinical trials of this recombinant fusion molecule in both adult melanoma and pediatric neuroblastoma patients have demonstrated its safety in patients at doses and schedules that are able to induce immune activation [183,184].

### 7.2. Recombinant Viruses as Delivery Systems for Tumor Immunotherapy

The expression of cytokines by recombinant viruses provides another strategy for increasing the immunogenicity of antigen-specific vaccines and for local delivery of cytokines to the tumor microenvironment. An attenuated oncolytic herpes simplex virus type 1 encoding GM-CSF was shown to selectively replicate in tumor cells, leading to the production of local GM-CSF with the potential to augment tumor-specific immunity. The virus was attenuated by deletion of pathogenic viral genes, leading to enhanced replication of the virus in tumor cells through increased expression of the herpes US11 promoter and to increased antigen presentation in HSV-infected cells. Local GM-CSF enhances DC uptake of necrotic tumor cells, maturation and induction of T cell immunity. Phase I studies demonstrated an acceptable safety profile with low-grade fever as the major side effect [185]. A Phase II multi-institutional clinical trial tested the vector by direct injection into accessible melanoma lesions in patients with unresectable Stage IIIc or IV melanoma [186]. Fifty patients were treated, and a 26% overall objective response rate (that included some regressions outside of the injected metastases) was reported. There was a correlation between clinical regression and increased MART-1-specific CD8+ T cells and a decrease in CD4+FoxP3+ Tregs at the tumor site [187]. Based on these results, a prospective, randomized Phase III clinical trial of the virally-encoded cytokine injected intralesionally compared with subcutaneous administration of GM-CSF in patients with metastatic melanoma is currently underway [188].

Recombinant vaccinia viruses have also shown promise against a variety of tumors using *in vivo* murine tumor models. When tumors were injected directly with the oncolytic vector vaccinia virus, there was significant local cytokine production by dendritic cells and T cells [189,190]. Vaccinia virus-induced inhibition of T cell proliferation was seen but could be reversed by adding IL-2 and IL-12 to the vaccinia constructs, and the vaccinia-cytokine strategy led to profound local tumor regression [191]. This suggests that intratumoral vaccinia-cytokine gene constructs can retard tumor growth by targeting the immune system through tumor-infiltrating DC and T cells.

### 7.3. Cell Engineering Approaches

Genetic engineering of tumor cells, antigen-presenting cells and effector lymphocytes is being developed as a way of delivering cytokines into the tumor microenvironment in an optimally defined space and time to prime host anti-tumor immunity against natural tumor antigens that have been processed and presented in immunogenic fashion [192]. Improvements in *in vitro* technology have facilitated the identification of tumor antigens and the isolation and expansion of antigen-reactive T cells. In these approaches, whole irradiated autologous or allogeneic tumor cells have been shown to secrete cytokine for sufficient periods of time to prime effective immune responses, and the possibility of introducing genes encoding accessory molecules not ordinarily expressed by the tumor has also been explored. Cytokines secreted locally in this fashion can also provide proliferative and survival signals to antigen-presenting and effector cells that further promote immune responses [193]. It has been shown that irradiated tumor cells in a vaccine expressing murine GM-CSF have been demonstrated to stimulate potent, long-lasting, and specific anti-tumor immunity, requiring both CD4+ and CD8+ cells [194]. Clinical trials of allogeneic irradiated whole cell tumor vaccines encoding GM-CSF have been reported, with the theoretical potential that an allogeneic source of such an engineered tumor cell vaccine could provide an “off the shelf” consistent and well-characterized source of the therapeutic agent, but the best overall choice of genetically-engineered tumor cell vaccine remains to be determined [195].

### 7.4. Cytokine PEGylation

Conjugation of the polymer polyethylene glycol (PEG) to proteins is termed PEGylation. This process can significantly decrease protein clearance from plasma and increase the *in vivo* half-life, providing a method for enhancing exposure to specific proteins and potentially avoiding toxicities associated with high peak concentrations of the unmanipulated protein [196]. To date, PEGylation has been successfully applied to two cytokines: PEG-interferon-alpha-2a (PEG-IFN-α2a) and PEG-granulocyte colony-stimulating factor (PEG-G-CSF). IFN-α2a and PEG-IFN-α2a are virtually indistinguishable, and both formulations elicit IFN response genes with equal efficiency while inhibiting tumor development with equal potency [197]. However, PEG-IFN-α2a has a more favorable safety profile and greater convenience associated with less frequent dosing, and largely for this reason, it was recently approved as adjuvant treatment for stage III melanoma. While the use of standard interferon as adjuvant therapy remains controversial due to the disparity between its high toxicity and minimal benefit, the approval of PEG-interferon offers a slightly better toxicity-benefit ratio.

## 8. Role of Cytokines in Vaccine and Adoptive Cell Therapies

In vaccination and adoptive cell strategies for cancer, cytokines play a dual role. They are critical for the *ex vivo* generation of the cell populations used in vaccines and adoptive cell therapy, and they are also important *in vivo* as adjuvants to these therapies to augment the potency and duration of anti-tumor response.

### 8.1. Vaccine Therapy

While cancer treatment vaccines have shown only modest activity with simpler regimens, an area of intense focus has been the use of cytokines as adjuvants to augment the immune response elicited by the vaccine [195,198]. The Cytokine Working Group conducted a study of high-dose IL-2 plus an HLA-A2-restricted gp-100 peptide in HLA-A2-positive patients with metastatic melanoma. The initial results have been mixed [199-201], and current therapies that appear to have a superior therapeutic index and to be more widely available (not requiring a specific HLA type as peptide vaccines do) are likely to temper enthusiasm for this approach.

Dendritic cell (DC)-based vaccination therapy for cancer is a very promising strategy, since DCs are the most potent T cell activators. Cytokines are used in several aspects of dendritic cell-based vaccine strategies, including their elicitation from peripheral blood monocytes obtained by leukapheresis (most commonly with IL-4 and GM-CSF) and their maturation to potent antigen-presenting cells (for example, with TNFα and IL-1β) [20]. The safety of using autologous DC vaccines has been reported in clinical trials enrolling over 1,000 cancer patients exposed to a wide variety of types of DC product, route and schedule of administration [202]. The only cancer vaccine that has been approved thus far— sipuleucel-T for prostate cancer—relies on the fusion of a prostate cancer antigen to GM-CSF, which is then loaded into autologous peripheral blood monocytes thought to be predominantly dendritic cells. The “built-in” GM-CSF provides a way to activate the dendritic cells away from the cancer's immunosuppressive microenvironment so the dendritic cells can then present the cancer antigen to the T cells and elicit an immune response.

A similar approach to incorporate cytokines into vaccine therapy has been to transfect tumor cells used in vaccination with the gene for cytokines such as IL-2 or GM-CSF to localize the cytokine effects to the sites of tumor and T cell activation.

### 8.2. Adoptive Cell Therapy

The scope of cytokine biology is perhaps most elaborately showcased by adoptive cell therapy (ACT). ACT involves the development and expansion of a patient's anti-tumor T cells outside of the body, away from the suppressive and inhibitory signals of a cancer microenvironment, followed by the reinfusion of these cells back into the patient to exert an anti-tumor response. The main advantage of ACT over less technical forms of immunotherapy is the control and precision it allows in creating— through the use of cytokines—an ideal, highly-manipulated environment to optimize the development of anti-tumor T cells. After a patient's blood product is obtained by leukapheresis, cytokines such as GM-CSF, IL-4, TNF-α, IL-6, IL-1β are used in the development and activation of their dendritic cells, and cytokines such as IL-2, IL-7, and more recently, IL-21 and IL-15, are used in the stimulation and expansion phase of T cells. In addition to the use of *ex vivo* cytokines in the generation of T cells, the administration of low-dose IL-2 to the patient following T cell infusions has been found to enhance the *in vivo* survival of the adoptively transferred cells, which is highly correlated with clinical outcome [203]. Thus, a course of IL-2 following T cell transfer and has become a standard component of ACT.

Another strategy that has been found to enhance adoptive therapy is the addition of a lymphodepleting regimen prior to the transfer of anti-tumor T cells. In a comparison analysis of three of their clinical trials that used increasingly myeloablative conditioning regimens, the NCI Surgery Branch found a strong positive correlation between response rate and the intensity of lymphodepletion [204]. One of the main reasons creating a lymphopenic environment prior to T cell infusion is thought to be so critical is the reduction in competition for cytokines from other lymphocytes and the increase in homeostatic cytokines IL-7 and IL-15.

While IL-2 has generally been utilized to maintain persistence of adoptively transferred T cells, there is evidence that IL-7 and IL-15 may be superior to IL-2 due to a more favorable profile in preferentially maintaining memory CD4+ and CD8+ T cells over CD4+CD25+Foxp3+ regulatory T cells [93,205]. IL-21 has also been found to enhance the *ex vivo* expansion, phenotype and affinity of antigen-specific T cells used in adoptive cell therapy. Further studies are ongoing to better understand the optimal use of these cytokines in T cell persistence, migration and homeostatic repopulation to improve therapeutic effectiveness of adoptive T cell approaches.

## 9. Conclusions

The generation of potent, specific, and durable anti-tumor immunity requires a variety of cytokines that regulate important functions related to the balance between tumor rejection by antigen-specific effector cells and suppressive mechanisms that allow tumors to escape immunologic detection. The cytokines are critical for tumor immunosurveillance and have demonstrated therapeutic anti-tumor activity in murine models and in the clinical treatment of several human cancers. Single-agent IFN-α and high-dose IL-2 have been approved in the treatment of melanoma and renal cell carcinoma. Other members of the IL-2-related cytokine family are under intense investigation for additional anti-tumor applications based on encouraging murine tumor models. In addition, several innovative strategies have been developed that utilize cytokines to promote effective anti-tumor immunity, including bifunctional molecules such as antibody-cytokine fusions, expression of cytokines in recombinant viral vectors, or irradiated whole tumor cells as vaccines, by PEGylation to enhance the kinetics, and for *ex vivo* manipulation of cells, such as dendritic cells and adoptively transferred T cells.

A better understanding of the molecular signaling pathways used by cytokine receptors and the temporal and kinetic pattern of receptor expression will be critical in the ongoing development of effective cytokine-based cancer treatments. Given the low response rates and significant toxicities of IL-2 and IFN-α, an important direction of additional research is the search for predictive biomarkers to improve the selection of patients most likely to respond. The extensive pleiotropy and redundancy of cytokine signaling suggests that the future of cytokine-based therapy may be in combination regimens that target multiple pathways to amplify the anti-tumor response while suppressing the regulatory pathways, and while minimizing toxicities. The recent advances in molecularly targeted therapies, such as BRAF inhibition, are already generating enthusiasm for a novel application of cytokines in combination with these therapies. Cytokines have proven to be effective in the treatment of cancer and there is little doubt they will continue to play a major role in the development of cancer immunotherapy.

## Figures and Tables

**Table 1.** General features of cytokines.

**Cytokine**	**Primary Cell Source**	**Primary Target Cell**	**Biological Activity**

GM-CSF	T cells Macrophages Endothelial cells Fibroblasts Mast cells	Bone marrow progenitor cells DC Macrophages NKT cells	Promotes antigen presentation T cell homeostasis Hematopoietic cell growth factor
IL-1	Monocytes Macrophages Fibroblasts Epithelial cells Endothelial cells Astrocytes	T cells B cells Endothelial cells Hypothalamus Liver	Co-stimulation Cell activation Inflammation Fever Acute phase reactant
IL-2	T cells NK cells	T cells NK cells B cells Monocytes	Cell growth/ activation
IL-3	T cells	Bone marrow progenitor cells	Cell growth and differentiation
IL-4	T cells	T cells B cells	Th2 differentiation Cell growth/activation IgE isotype switching
IL-5	T cells	B cells Eosinophils	Cell growth/ activation
IL-6	T cells Macrophages Fibroblasts	T cells B cells Liver	Co-stimulation Cell growth/ activation Acute phase reactant
IL-7	Fibroblasts Bone marrow stromal cells	Immature lymphoid progenitors	T cell survival, proliferation, homeostasis B cell development
IL-8	Macrophages Epithelial cells Platelets	Neutrophils	Activation Chemotaxis
IL-10	Th2 T cells	Macrophages T cells	Inhibits antigen-presenting cells Inhibits cytokine production
IL-12	Macrophages NK cells	T cells	Th1 differentiation
IL-15	Monocytes	T cells NK cells	Cell growth/ activation NK cell development Blocks apoptosis
IL-18	Macrophages	T cells NK cells B cells	Cell growth/ activation Inflammation
IL-21	CD4+ T cells NKT cells	NK cells T cells B cells	Cell growth/ activation Control of allergic responses and viral infections
IL-23	Antigen-presenting cells	T cells NK cells DC	Chronic inflammation Promotes Th17 cells
IFN-α	Plasmacytoid DC NK cells T cells B cells Macrophages Fibroblasts Endothelial cells Osteoblasts	Macrophages NK cells	Anti-viral Enhances MHC expression
IFN-γ	T cells NK cells NKT cells	Monocytes Macrophages Endothelial Cells Tissue cells	Cell growth/ activation Enhances MHC expression
TGF-β	T cells Macrophages	T cells	Inhibits cell growth/activation
TNF- α	Macrophages T cells	T cells B cells Endothelial cells Hypothalamus Liver	Co-stimulation Cell activation Inflammation Fever Acute phase reactant

DC—dendritic cell; GM-CSF—granulocyte-macrophage colony stimulating factor; IL—interleukin; IFN—interferon; TNF—tumor necrosis factor;TGF—transforming growth factor.

**Table 2.** Classification of cytokine receptor families.

**Receptor Family**	**Ligands**	**Structure/Function**

Type I CytokineReceptors	IL-2 IL-3 Il-4 IL-5 IL-6 IL-7 IL-9 IL-11 IL-12 IL-13 IL-15 IL-21 IL-23 IL-27 Erythropoietin GM-CSF G-CSF Growth hormone Prolactin Oncostatin M Leukemia inhibitory factor	Composed of multimeric chains. Signals through JAK-STAT pathway using common signaling chain. Contains cytokine binding chains.
Type II Cytokine Receptors	IFN-α/β IFN-γ IL-10 IL-20 IL-22 IL-28	Immunoglobulin-like domains. Uses heterodimer and multimeric chains. Signals through JAK-STAT.
Immunoglobulin Superfamily Receptors	IL-1 CSF1 c-kit IL-18	Shares homology with immunoglobulin structures.
IL-17 Receptor	IL-17 IL-17B IL-17C IL-17D IL-17E IL-17F	
G Protein-Coupled Receptors (GPCR)	IL-8 CC chemokines CXC chemokine	Functions to mediate cell activation and migration.
TGF-β receptors 1/2	TGF-β	

Tumor Necrosis Factor Receptors (TNFR)	CD27 CD30 CD40 CD120 Lymphotoxin-β	Functions as co-stimulatory and co-inhibitory receptors.

CD—cluster of differentiation; c-kit—mast/stem cell growth factor receptor; CSF—colony-stimulating factor; G-CSF—granulocyte-colony stimulating factor; GM-CSF—granulocyte-macrophage colony stimulating factor; IL—interleukin; JAK—janus kinase; STAT—signal transducer and activator of transcription; TGF—transforming growth factor.
